# Seed mucilage in temperate grassland species is unrelated to moisture requirements

**DOI:** 10.1002/pei3.10135

**Published:** 2024-02-21

**Authors:** Laura M. Ladwig, Jessica R. Lucas

**Affiliations:** ^1^ Biology Department University of Wisconsin Oshkosh Wisconsin USA

**Keywords:** functional trait, grassland, mucilage, myxocarpy, myxodiaspory, myxospermy, prairie, seed ecology

## Abstract

Myxospermy, the release of seed mucilage upon hydration, plays multiple roles in seed biology. Here, we explore whether seed mucilage occurs in a suite of temperate grassland species to test if the prevalence of species producing seed mucilage is associated with habitat type or seed characteristics. Seventy plant species found in wet or dry North American temperate grasslands were tested for the presence of seed mucilage through microscopic examination of seeds imbibed with histochemical stain for mucilage. Mucilage production was compared among species with different moisture requirements and seed mass. In this study, 43 of 70 of species tested produced seed mucilage. Seed mucilage did not differ based on habitat type, species moisture requirements, or seed mass. Most seed mucilage was non‐adherent and did not remain stuck to the seed after extrusion. Seed mucilage was a common trait in the surveyed temperate grassland species and was observed in 61% of evaluated species. Surprisingly, seed mucilage was more common in temperate grasslands than in previous ecological surveys from arid/semiarid systems, which found 10%–31% myxospermous species. Given the high prevalence, seed mucilage may influence seedling ecology in temperate grasslands and requires further investigation.

## Graphical abstract

Seeds exude mucilage upon hydration to help multiple aspects of seedling physiology and ecology. This paper reports over 60% of grassland species produce mucilage.

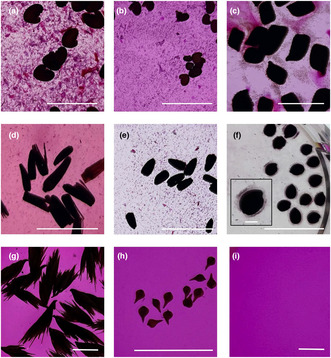

## 1 INTRODUCTION

All species possess adaptive traits, and understanding such traits is helpful to maintain biodiversity and mediate impacts of climate change. Many plant species exude mucilage from their seeds and/or fruit upon contact with water (Grubert, 1981). This mucilage is a heterogenous mixture of carbohydrates including pectins and hemicelluloses (Kreitschitz, 2009; Tsai et al., 2021; Viudes et al., 2020; Western, 2012; Yang, Baskin, Baskin, & Huang, 2012; Yang, Baskin, Baskin, Liu, et al., 2012). Regardless of whether mucilage was expelled from the seed or fruit (myxospermy or myxocarpy, respectively), it is considered an adaptive trait that impacts multiple aspects of seed ecology (Phan & Burton, 2018; Western 2014; Yang, Baskin, Baskin, & Huang, 2012). Within a plant community, seed mucilage may influence species persistence and shape community composition through different aspects of seed biology (Archibold, 1995; Steiger, 1930; Werling et al., 2014).

Seed mucilage is a common trait in angiosperms, and it appears to play roles in seed survival, dispersal, and germination (Fernandez‐Alonso et al., 2003; LoPresti et al., 2022; LoPresti et al., 2023; Tsai et al., 2021, Western, 2012; Yang, Baskin, Baskin, & Huang, 2012). Seed mucilage has been associated with dry and semi‐arid environments (LoPresti et al., 2022; Yang, Baskin, Baskin, & Huang, 2012). Surveys of plant communities in arid and semi‐environments indicate that 10%–32% of species produce seed mucilage (Table 1 and references therein). In dry, arid, and sandy environments, mucilage is proposed to adhere seeds to their surroundings and possibly retain water (Kreitschitz, 2009; LoPresti et al., 2023). Focused studies have shown species‐specific impacts of mucilage on seed ecology. Seed mucilage influences germination rates and timing in species‐specific manners (Bhatt et al., 2016; Geneve et al., 2017; Hu, Baskin, et al., 2019; Hu, Zhang, et al., 2019; Huang & Gutterman, 1999; Mascot‐Gómez et al., 2020; Teixeira et al., 2020; Witztum et al., 1969; Yang, Baskin, Baskin, Liu, et al., 2012; Zhao et al., 2020; Zhou et al., 2022). Seed mucilage has been demonstrated to anchor seeds to soil (Fuller and Hay, 1983; LoPresti et al., 2019; LoPresti et al., 2023), prevent desiccation (Teixeira et al., 2020), mediate salt stress (Huang & Gutterman, 1999), camouflage and protect seed from herbivores (LoPresti et al., 2019; LoPresti et al., 2022; LoPresti et al., 2023; Pan et al., 2021; Stessman et al., 2023), and support dispersal (Ronsted et al., 2002; Viudes et al., 2020). As mucilage impacts multiple aspects of seed ecology (LoPresti et al., 2023), the presence of mucilage could influence the establishment of some species over others within communities. As temperate grasslands are critical habitats in the United States, we seek to characterize the presence of seed mucilage in prairie species as a step toward aiding prairie restoration.

**TABLE 1:** Prevalence of myxospermy found in other community assessments.

Myxospermous species	Ecosystem/habitat type	Location	Reference
11.3% (66 of 582)	Broken veld	Namaqualand, South Africa	van Rooyen et al. (1990)
13.7% (7 of 51)	Mediterranean thorn cushion plant formation	High Atlas National Park; Tirrhist, Morocco	Navarro, El Oualidi, et al. (2009)
15.7% (22 of 140)	Semiarid shrublands	Cabo de Gata Natural Park, SE Spain	Navarro, Pascual, et al. (2009)
19.8% (14 of 72)	Semiarid grassland/semiarid desert	Southern Spain	Hensen (1999)
21.7% (64 of 296)	Hyper‐arid hot desert (including salt flats, gravel plains, sand sheets, and the Hajar Mountains)	United Arab Emirates	Navarro et al. (2021)
10.1%–27%^a^ (296 total species)	Multiple Semiarid roadsides	East Spain	Bochet and García‐Fayos (2015)
22.5%–31.4%^a^ (139 total species)	Multiple Semiarid shrublands	Spain	García‐Fayos et al. *(* 2013)

^a^ Multiple habitats and reported percentages for each.

Grasslands contain high amounts of biodiversity in relatively small areas (Werling et al., 2014), and the current coverage of grasslands in the Midwestern USA is only a slight faction of its historical range (Bolliger et al., 2004). To combat habitat loss, grasslands are being reintroduced in many regions through restoration actions, yet outcomes of grassland restorations can be quite variable and unpredictable (Brudvig et al., 2017; Norland et al., 2018). Planting seeds is a common grassland restoration technique (Smith et al., 2010), and an enhanced understanding of seed ecology related to establishment success is needed to improve the success and predictability of restoration efforts (Török et al., 2021). Seed mucilage could influence establishment and persistence of species in grassland restorations. Although seed mucilage has been catalogued in many angiosperm families common to grasslands, including Asteraceae, Brassicaceae, Fabaceae, Lamiaceae, and Poaceae (Grubert, 1981; Viudes et al., 2020; Yang, Baskin, Baskin, & Huang, 2012), the occurrence of mucilage in temperate grassland communities has not yet been documented. Grasslands occur across a range of abiotic and climatic stresses including dry sand prairies to wet meadows (Blair et al., 2014; Curtis, 1959), so the importance and prevalence of seed mucilage could vary across this moisture gradient.

Here, we evaluated the presence of seed mucilage in 70 plant species from wet and dry prairie restoration seed mixes to address several research questions. First, what is the prevalence of seed mucilage in temperate grasslands? Based on previous studies examining the abundance of seed mucilage within plant communities (Table 1), we predicted seed mucilage in less than one third of species. Second, does the prevalence of seed mucilage differ with moisture availability? We predicted a higher occurrence of seed mucilage in dry grassland species relative to wetter grassland species given the beneficial roles of mucilage in seed ecology in dry environments (Bhatt et al., 2016; Teixeira et al., 2020; Zhao et al., 2020). Lastly, does seed mucilage relate to other seed characteristics? As mucilage and mass are both energy‐rich investments, we hypothesized that seed mucilage would occur in grassland species with larger seed mass.

## 2 METHODS

### 2.1 Mucilage detection

We assessed the presence and persistence of seed mucilage in 70 prairie species (see “Section 2.2: Species Selection” below). Mucilage can be challenging to detect because it is often transparent and appears transiently after seed hydration. To easily visualize mucilage, seeds were incubated in an aqueous ruthenium red solution to stain pectins, the main polysaccharide of mucilage, purple (McFarlane et al., 2014). Twenty seeds of each species were incubated for at least 24 h in individual compartments of 6‐well plates with 2 mL of 0.01% ruthenium red dye in 50 mM EDTA pH 8.0 in the dark without shaking (McFarlane et al., 2014). The EDTA buffer chelates calcium ions thereby facilitating the release and expansion of pectins from the seed into the surrounding media (McFarlane et al., 2014). Stained seeds and mucilage were digitally photographed on a Leica S9 stereomicroscope with transmitted white light within the clear plastic 6‐well plates at 10 min, 1, 2, and 24 or 27 h. Negative control wells contained ruthenium red stain and no seeds to account for any ambient color changes or stain precipitation over the time course of the experiment.

The presence or absence of mucilage was obvious after ruthenium red staining for most species. Seed images were independently examined by two researchers to detect ruthenium red stained mucilage. Seed mucilage characteristics varied among species, and we classified mucilage into three categories based on visualizations with ruthenium red: adherent, non‐adherent, and both adherent and non‐adherent. Adherent mucilage remained attached to seed coats after 24 h. Non‐adherent mucilage sluffed off, separated from seeds, and floated in the surrounding media. For a small number of species, the presence of mucilage was not easily determined from images or was inconsistent between observers, and so these species were retested. Most of these species were small‐seeded, and so additional trials were performed in small‐welled (24 well) plates with less dye solution (1 mL) to prevent dilution of the mucilage into the dye solution and aid detection.

### 2.2 Species selection

Hundreds of plant species can be found in North American prairies (Steiger, 1930), and we chose a subset of species with preferences across a range of wet to dry environments to test if seed mucilage varied with moisture requirements. The species selected represent a range of abundances found at Midwestern prairie sites based on historical prairie surveys in Wisconsin (Curtis, 1959, Henderson, 1998). For example, our study species ranged from dominant grasses (e.g., *Andropogon gerardii*, *Schizachyrium scoparium*) to uncommon forbs (e.g., *Callirhoe triangulata*, *Penstemon grandiflorus*). All seeds were purchased from a native species seed supplier that provides regional genotypes (Prairie Moon Nursery, Winona, MN USA, www.prairiemoon.com). Seeds were collected by the supplier in Illinois, Iowa, Minnesota, and Wisconsin sites.

Selected species were from either a dry or wet prairie seed mix. The dry seed mix (Shortgrass Dry Sand Prairie Seed Mix) contained 35 species that ranged in moisture requirements from dry to mesic, and the wet seed mix (Tallgrass Prairie Seed Mix for Medium‐Wet Soil) contained 36 species that ranged in moisture requirements from mesic to wet (Table S1). Only one species was included in both seed mixes (*Rudbeckia hirta* L.). Additionally, the seed supplier provided moisture requirement categories for each species (dry, dry‐mesic, mesic, wet‐mesic, wet) which allowed for comparison between mucilage production and moisture requirements at a finer, species‐specific scale. We confirmed the moisture requirements listed by the seed supplier with the USDA Plants Database (USDA NRCS, 2023). Moisture classifications were consistent between data sets, but the USDA Plant Database did not have detailed information for all the study species, so moisture classifications from the seed supplier were used for statistical analysis.

### 2.3 Statistical analysis

To statistically evaluate differences in seed mucilage occurrence in dry and wet communities, we compared mucilage presence/absence between the two seed mixes with a contingency table and chi‐squared test. Furthermore, we tested if seed mucilage in each of the five detailed moisture classifications differed from an expected random distribution with a contingency table and chi‐squared test.

To test whether seed mass differed among myxospermous and non‐myxospermous species in the two seed mixes we used seed mass provided by the seed supplier and performed an ANOVA to test if seed mass differed between myxospermous and non‐myxospermous species, and if it differed between seed mixes (lm(log(mass)) ~ Mucilage presence*SeedMix). All statistical tests were run using the “vegan” package in R (version 4.2.0, R Core Team, 2021) with an alpha of 0.05.

## 3 RESULTS

### 3.1 Seed mucilage present in majority of species

The presence and appearance of seed mucilage differs between species (Yang, Baskin, Baskin, & Huang, 2012, Figure 1), and we considered a species myxospermous if mucilage was visible with ruthenium red staining using a low‐magnification microscope. We imaged stained seeds at multiple time points after adding liquid (10 min, 1, 2, and 24 or 27 h). When present, mucilage emerged from most seeds within 1 h and remained visible throughout the experiment. We classified species as myxospermous regardless of when mucilage appeared during the 24 h experiment. In total, 43 of 70 (61%) grassland species in this survey produced seed mucilage (Table 2, Table S1).

**FIGURE 1:**
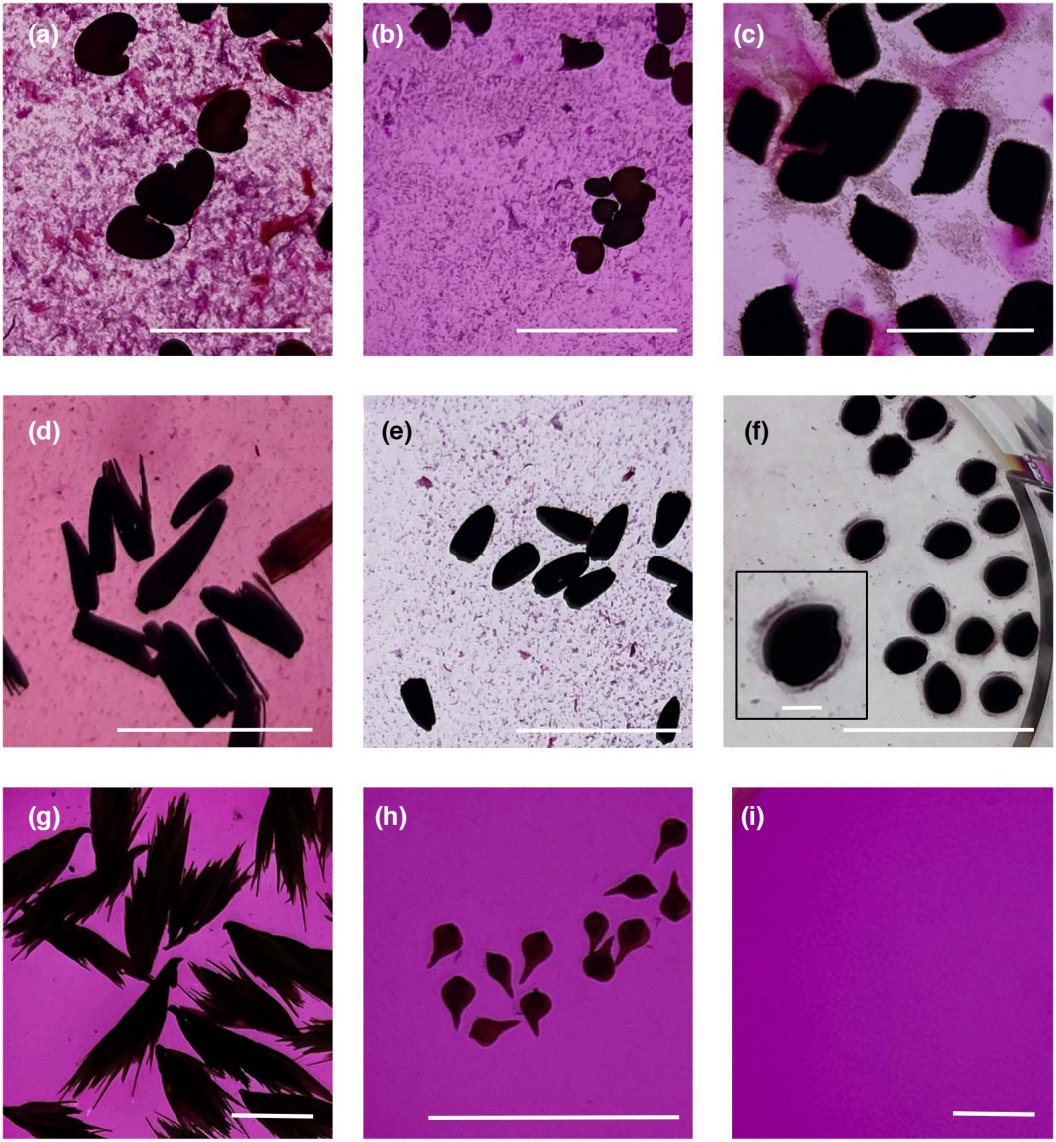
Majority of seeds exuded mucilage. Seeds (dark objects) hydrated and stained with ruthenium red for 24 h and visualized with transmitted light. (a) *Crotalaria sagittalis*, (b) *Dalea pupurea*, (c) *Chamaecrista fascicula*, (d) *Liatris pycnostachya*, (e) *Helianthus occidentalis*, (f) *Euphorbia corollate*, (g) *Bouteloua curtipendula*, (h) *Carex vulpinoides*, (i) Ruthenium red liquid control with no seeds. Species (a–f) produced mucilage (Stained dark purple), while (g) and (h) did not. The purple color of liquid became transparent after incubation with seeds (e) and (f) after 24 h. Inset in (f) showed adherent mucilage stained lightly pink. All magbars represent 10 mm, except 1 mm magbar in inset F.

**TABLE 2:** Myxospermy in surveyed grassland families.

Families	Species surveyed	Myxospermous
Asteraceae	19	18
Cypraceae	10	0
Poaceae	9	1
Fabaceae	8	5
Lamiaceae	3	3
Apocynaceae	2	1
Malvaceae	2	1
Verbenaceae	2	2
Apiaceae	1	0
Campanulaceae	1	1
Commelinaceae	1	0
Euphorbiaceae	1	1
Gentianaceae	1	1
Hypericaceae	1	1
Iridaceae	1	1
Juncaceae	1	1
Liliaceae	1	1
Onagraceae	1	1
Orobanchaceae	1	1
Plantaginaceae	1	1
Ranunculaceae	1	0
Rhamdaceae	1	1
Rosaceae	1	1
Total	70	43

**FIGURE 2:**
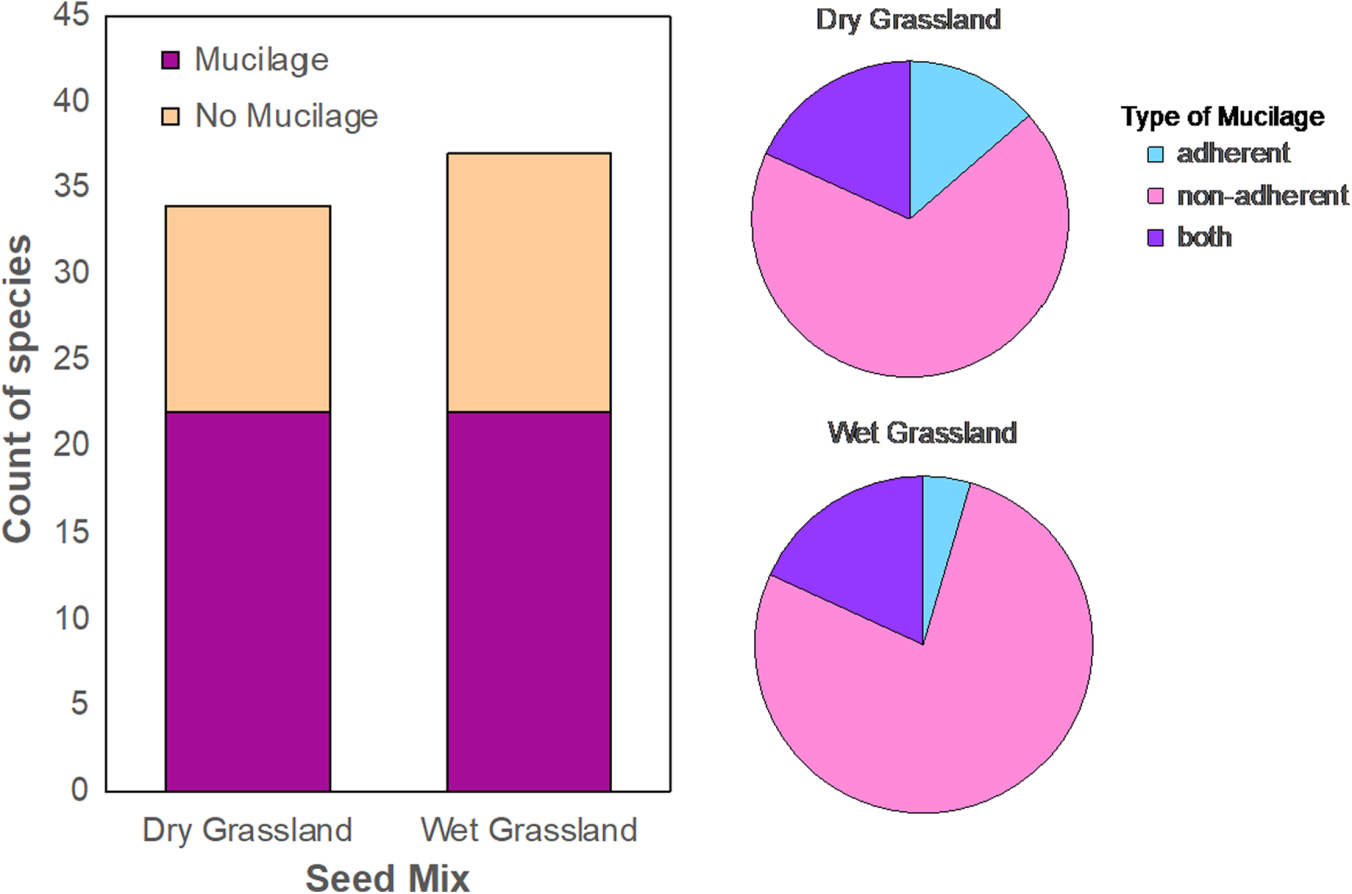
Prevalence of seed mucilage production for species in the two contrasting seed mixes (left). Proportions of the type of seed mucilage found in each grassland community type, adherent (blue, top right), non‐adherent (pink, bottom) or both adherent and non‐adherent (dark purple, top left). Both habitat types had the same number of myxospermous species (22), and the proportion of myxospermous species did not differ between mixes (*p* = .5).

The amount and type of mucilage varied among species (Figure 1; Table S1). For 32 species, mucilage was non‐adherent to the seed coat and diffused into the surrounding liquid. This non‐adherent mucilage appeared as deeply stained purple specks, wisps, or flakes in the surrounding liquid (Figure 2a‐e). In contrast, adherent mucilage remained attached to seeds throughout the 24 h of the experiment for four species (Figure 2f). Seeds of eight species exuded both adherent and non‐adherent mucilage (Table S1). While the type of mucilage varied among species, it appeared consistent within a species; we did not observe mucilage variation within seeds of single species. Adherent and non‐adherent mucilage may serve different roles for the plant by impacting the soil environment differently (Viudes et al., 2020).

Our species pool contained 23 families, and the families with highest representation included Asteraceae (19 species), Cypraceae (10 species), Poaceae (nine species), and Fabaceae (eight species; Table S1). Three or fewer species represented the remaining 19 families. In our survey, mucilage was common in Asteraceae (18 of 19 species) and Fabaceae (five of eight species), and nearly absent from Poaceae and Cyperaceae (Table 2).

### 3.2 Seed mucilage, water requirements, and seed size

**FIGURE 3:**
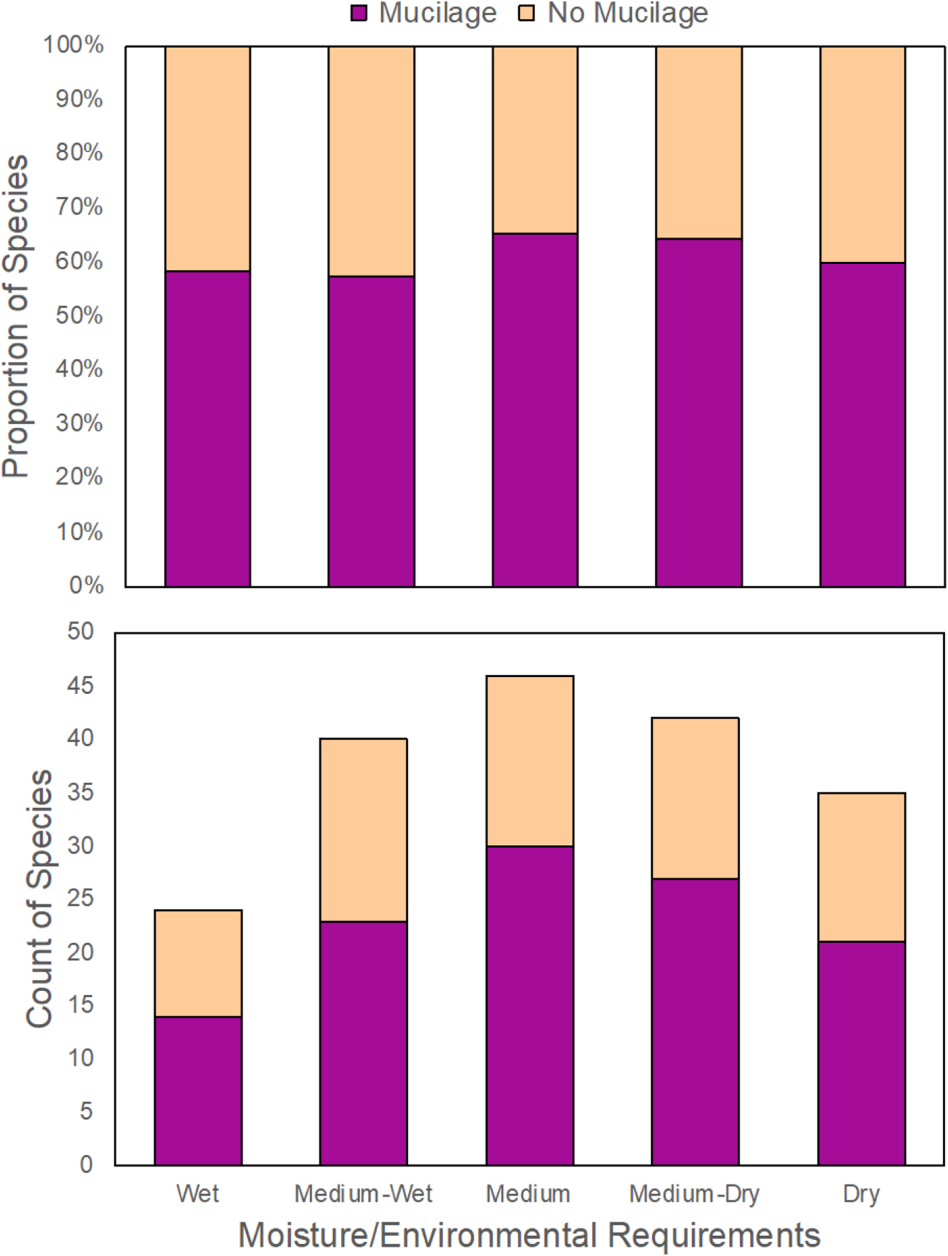
Proportion (top) and count (bottom) of species that produced seed mucilage (purple) or did not (light tan) for each moisture category. The proportion of myxospermous species did not statistically differ among the five moisture environments (*p* = .9).

**FIGURE 4:**
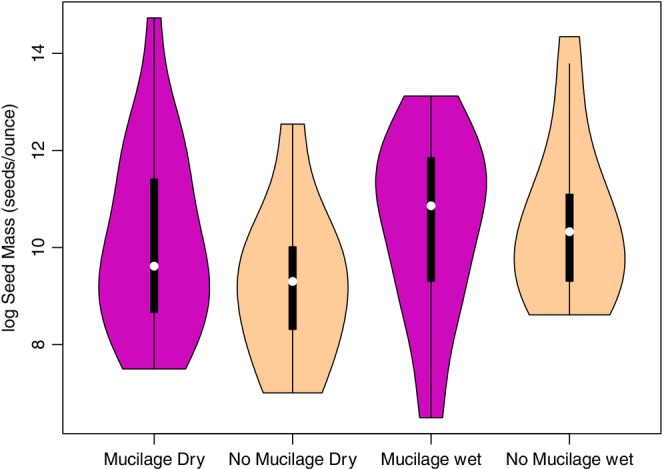
Distribution of seed mass for species that produced seed mucilage (purple) and species that did not (light tan) in the dry (left) and wet (right) grassland seed mixes. Seed mass was not statistically different between seed mixes or between myxospermous and non‐myxospermous species (*p* = .8).

We predicted a higher proportion of species would be myxospermous in the drier grassland, but the ratio of myxospermous species did not differ between wet and dry seed mixes (*p* = .5, *X* ^2^ = 0.4, df = 1; Figure 2). Additionally, when considering moisture requirements on a finer scale, the presence/absence of seed mucilage also did not differ among the five moisture categories (*p* = .9, *X* ^2^ = 0.9, df = 4, Figure 3). Lastly, we predicted that larger seeds would be more likely to produce seed mucilage, but seed mass did not differ among myxospermous and non‐myxospermous species or between the two seed mixes (*F*_3,65_ = 0.8, *p* = .5, Figure 4).

## 4 DISCUSSION

Myxospermy was much more prevalent in North American temperate grassland communities than expected, with almost two‐thirds of surveyed grassland species (61%) producing seed mucilage. Myxospermy may influence physiological and ecological processes in grasslands and has been catalogued in many angiosperm families common to grasslands, (e.g., Asteraceace, Brassicaceae, Fabaceae, Lamiaceae, and Poaceae; Grubert, 1981; Viudes et al., 2020; Yang, Baskin, Baskin, & Huang, 2012), yet the prevalence of myxospermous species in mesic grassland had not been evaluated previously. Seed mucilage is largely composed of pectin and other high‐energy carbohydrates that the plant could allocate elsewhere in the developing seed, so investing energy in seed mucilage presumably serves an important purpose or beneficial adaptation (LoPresti et al., 2022; Yang, Baskin, Baskin, & Huang, 2012). Given the high prevalence of myxospermy, seed mucilage presumably provides benefits for grassland species.

One benefit of seed mucilage with strong support in the literature is its ability to make seeds more drought tolerant by helping regulate osmotic stress (Geneve et al., 2017; Mascot‐Gómez et al., 2020; Teixeira et al., 2020; Zhao et al., 2020). Seed mucilage benefits plant performance for many dry‐adapted species (e.g., Bhatt et al., 2016; Mascot‐Gómez et al., 2020; Teixeira et al., 2020; Yang, Baskin, Baskin, Liu, et al., 2012), therefore, we predicted that dry‐adapted species would be more likely to produce seed mucilage than mesic or wet‐adapted species, but that was not the case. Instead, the percentage of species that produced seed mucilage was similarly high for both dry (65%) and wet (59%) seed mixes (Figure 2). Furthermore, when examining species using a finer gradient of moisture preferences, dry‐adapted species were equally likely to produce seed mucilage as wet‐adapted species (Figure 3). Although reducing osmotic stress is an important role of seed mucilage in arid/semiarid systems (Mascot‐Gómez et al., 2020; Teixeira et al., 2020), additional roles of seed mucilage beyond drought tolerance could be more influential in mesic to wet systems. Other ecological benefit of seed mucilage could include attracting beneficial symbionts (Geneve et al., 2017; Hu, Zhang, et al., 2019; Lukomets et al., 2020), adhering seeds to soil (Gutterman & Shem‐Tov, 1997; LoPresti et al., 2022), or deterring granivory (LoPresti et al., 2023; Pan et al., 2021). The specific ecological roles of seed mucilage in temperate grasslands remains unknown and requires further investigation.

Seed mucilage is an energy investment and larger seeds contain more energy, yet the production of seed mucilage was unrelated to seed mass across the grassland plant species tested here (Figure 4). Previous research has also found no association between seed size and mucilage production (Grubert, 1982). If mucilage production was constrained by resource availability, smaller seeds may exude less mucilage due to lower resource availability. Yet, myxospermy was equally present regardless of seed mass, so for these small‐seeded species the benefits of mucilage may outweigh the energetic costs. Mucilage can provide a useful adhesive to hold seeds in place to allow for germination (Gutterman & Shem‐Tov, 1997), and these adhesive benefits may be disproportionately more beneficial to smaller seeds than larger ones. Seed mass is correlated with many aspects of seed ecology (Díaz et al., 2016; Moles & Westoby, 2006) but does not relate to mucilage production for these grassland species.

We measured a much higher occurrence of myxospermy in temperate grasslands (61%) than previous assessments in arid/semiarid plant communities (10%–31%, Table 1). Differences in methodology could account for part of this difference. Previous mucilage surveys did not clearly describe detection protocols, and therefore we are unable to make direct comparisons or replicate previous surveys. As mucilage is often transparent and non‐adherent, it can be difficult to visualize without a histochemical stain and microscopy. Additionally, mucilage production is not always immediate and can occur over 24 h after seed hydration so rapid assays may exclude species with slow exuding mucilage. Regardless of possible differences in detection methods, most temperate grassland species tested here were myxospermous. Given the high prevalence of myxospermy encountered in our study, myxospermy may play a previously unexamined ecological role in more systems than we currently realize.

Now that we know so many grassland species make seed mucilage, the next logical step is to explore possible reasons. From an energetics perspective, expelling useful carbohydrates into the environment could be wasteful without some sort of reward or benefit to the germinating seed. There are many proposed ecological benefits of seed mucilage (LoPresti et al., 2022; Yang, Baskin, Baskin, & Huang, 2012), yet few have been tested outside of the laboratory in more natural settings (LoPresti et al., 2023). Additionally, we could start including seed mucilage in functional trait investigations (Saatkamp et al., 2019) to better understand how seed mucilage relates to species establishment and persistence in the variety of different communities where myxospermy is found. Myxospermy is not included in the methodological handbook for measuring ecological plant traits (Pérez‐Harguindeguy et al., 2016) and has limited coverage in a widely used global trait databases; as of April 2022, there was only one regional survey that included myxospermy in the TRY Plant Trait database (Kattge et al., 2020).

We found a high occurrence of myxospermous species in two grassland plant assemblages but in general, we do not know the prevalence or ecological role of myxospermy across terrestrial ecosystems. Staining for seed mucilage production is a relatively straightforward and economical process that could be added to the suite of functional traits commonly measured by ecologists and botanists. Additionally, more extensive surveys of the presence of myxospermy in a variety of ecosystems paired with experiments are needed to understand the full ecological extent of seed mucilage.

## CONFLICT OF INTEREST STATEMENT

The authors declare that they have no conflict of interest.

## Supporting information


Table S1.

## Data Availability

All data used in statistical analysis are included in Table S1.
