# Supplementary material for: Seed mucilage in temperate grassland species is unrelated to moisture requirements
Source: Plant Environ Interact. 2024 Feb 21;5(1):e10135. doi: 10.1002/pei3.10135 (PMC10880130; doi:10.1002/pei3.10135)
Supplement: Supplementary file 1 — Table S1. [file PEI3-5-e10135-s001.docx]

Supplemental Table 1: Species, and their characteristics, included in this study. In the “Seed Mucilage Present” column, superscripts a and b refer to adherent and both adherent and non-adherent mucilage respectively. All other myxospermous species produced non-adherent mucilage.

| **Family** | **Species** | **Seed Mucilage present** | **Seed mass (seed/oz)** | **Seed Mix** | **wet** | **med-wet** | **medium** | **med-dry** | **dry** |
| --- | --- | --- | --- | --- | --- | --- | --- | --- | --- |
| Apiaceae | *Zizia aurea* (L.) W.D.J. Koch | No | 11000 | Wet |  | x | x | x |  |
| Apocynaceae | *Asclepias incarnata* L. | Yes | 4800 | Wet | x | x | x |  |  |
| Apocynaceae | *Asclepias tuberosa* L. | No | 4300 | Dry |  |  | x | x | x |
| Asteraceae | *Artemisia ludoviciana* Nutt. | Yes | 275000 | Dry |  |  | x | x | x |
| Asteraceae | *Coreopsis lanceolata* L. | No | 14000 | Dry |  |  |  | x | x |
| Asteraceae | *Echinacea pallida*(Nutt.) Nutt. | Yes | 5200 | Dry |  |  | x | x | x |
| Asteraceae | *Eupatorium perfoliatum* L. | Yes | 160000 | Wet | x | x |  |  |  |
| Asteraceae | *Eutrochium maculatum* (L.) E.E. Lamont | Yes | 80000 | Wet | x | x |  |  |  |
| Asteraceae | *Helenium autumnale* L. | Yes | 140000 | Wet | x | x |  |  |  |
| Asteraceae | *Helianthus occidentalis* Riddell | Yes ^b^ | 14000 | Dry |  |  | x | x | x |
| Asteraceae | *Liatris pycnostachya* Michx. | Yes ^a^ | 11000 | Wet | x | x | x |  |  |
| Asteraceae | *Oligoneuron riddellii* (Frank ex Riddell) Rydb. | Yes | 80000 | Wet | x | x | x |  |  |
| Asteraceae | *Rudbeckia hirta* L. | Yes | 92000 | Both |  | x | x | x |  |
| Asteraceae | *Rudbeckia subtomentosa* Pursh | Yes | 52000 | Wet |  | x | x | x |  |
| Asteraceae | *Rudbeckia triloba* L. | Yes | 40000 | Wet |  | x | x | x |  |
| Asteraceae | *Silphium laciniatum* L. | Yes ^b^ | 660 | Wet |  | x | x | x | x |
| Asteraceae | *Silphium terebinthinaceum* Jacq. | Yes ^b^ | 1000 | Wet |  | x | x | x |  |
| Asteraceae | *Solidago nemoralis* Aiton | Yes | 250000 | Dry |  |  |  | x | x |
| Asteraceae | *Symphyotrichum novae-angliae* (L.) G.L. Nesom | Yes ^b^ | 80000 | Wet | x | x | x | x |  |
| Asteraceae | *Symphyotrichum oolentangiense* (Riddell) G.L. Nesom | Yes | 70000 | Dry |  |  | x | x | x |
| Asteraceae | *Symphyotrichum sericeum* (Vent.) G.L. Nesom | Yes | 32000 | Dry |  |  |  | x | x |
| Asteraceae | *Vernonia fasciculata* Michx. | Yes | 20000 | Wet |  | x | x |  |  |
| Campanulaceae | *Lobelia siphilitica* L. | Yes | 500000 | Wet | x | x | x |  |  |
| Commelinaceae | *Tradescantia ohiensis* Raf. | No | 8000 | Dry |  | x | x | x | x |
| Cypraceae | *Carex bebbii* Olney ex Fernald | No | 125000 | Wet |  | x | x |  |  |
| Cypraceae | *Carex brevior* (Dewey) Mack. | No | 29000 | Dry |  | x | x | x | x |
| Cypraceae | *Carex comosa* Boott | No | 37000 | Wet | x | x |  |  |  |
| Cypraceae | *Carex hystericina* Muhl. ex Willd. | No | 30000 | Wet | x | x |  |  |  |
| Cypraceae | *Carex molesta* Mack. ex Bright | No | 25000 | Dry |  | x | x | x | x |
| Cypraceae | *Carex stipata* Muhl. ex Willd. | No | 34000 | Wet | x | x |  |  |  |
| Cypraceae | *Carex vulpinoidea* Michx. | No | 80000 | Wet | x | x | x | x |  |
| Cypraceae | *Scirpus atrovirens* Willd. | No | 460000 | Wet | x | x |  |  |  |
| Cypraceae | *Scirpus cyperinus* (L.) Kunth | No | 1700000 | Wet | x | x |  |  |  |
| Cypraceae | *Scirpus validus* Vahl | No | 31000 | Wet | x | x |  |  |  |
| Euphorbiaceae | *Euphorbia corollata* L. | Yes ^a^ | 8000 | Dry |  |  | x | x | x |
| Fabaceae | *Amorpha canescens* Pursh | No | 16000 | Dry |  |  | x | x | x |
| Fabaceae | *Baptisia bracteata* Muhl. ex Elliott | No | 1700 | Dry |  |  | x | x | x |
| Fabaceae | *Chamaecrista fasciculata* (Michx.) Greene | Yes ^b^ | 2500 | Dry |  |  | x | x | x |
| Fabaceae | *Crotalaria sagittalis* L. | Yes | 3600 | Dry |  |  |  | x | x |
| Fabaceae | *Dalea candida* Michx. ex Willd. | Yes | 22000 | Dry |  |  | x | x | x |
| Fabaceae | *Dalea purpurea* Vent. | Yes | 15000 | Dry |  |  | x | x | x |
| Fabaceae | *Lupinus perennis* L. | No | 1100 | Dry |  |  |  | x | x |
| Fabaceae | *Tephrosia virginiana* (L.) Pers. | Yes ^b^ | 1800 | Dry |  |  | x | x | x |
| Gentianaceae | *Gentiana andrewsii* Griseb. | Yes | 280000 | Wet |  | x | x |  |  |
| Hypericaceae | *Hypericum pyramidatum* Aiton | Yes | 225000 | Wet |  | x | x |  |  |
| Iridaceae | *Iris virgninica* L. var. *shrevei* (Small) E.S. Anderson | Yes | 8000 | Wet | x | x | x |  |  |
| Juncaceae | *Juncus dudleyi* Wiegand | Yes ^a^ | 2500000 | Dry |  | x | x | x |  |
| Lamiaceae | *Monarda punctata* L. | Yes | 90000 | Dry |  |  |  | x | x |
| Lamiaceae | *Physostegia virginiana* (L.) Benth. | Yes | 15000 | Wet | x | x | x |  |  |
| Lamiaceae | *Pycnanthemum virginianum* (L.) T. Dur. & B.D. Jacks. ex B.L. Rob. & Fernald | Yes | 200000 | Wet | x | x | x | x |  |
| Liliaceae | *Melanthium virginicum* L. | Yes ^b^ | 5500 | Wet | x | x | x |  |  |
| Malvaceae | *Callirhoe triangulata* (Leavenworth) A. Gray | Yes | 5800 | Dry |  |  |  | x | x |
| Malvaceae | *Hibiscus laevis* All. | No | 2200 | Wet | x | x |  |  |  |
| Onagraceae | *Oenothera rhombipetala* Nutt. ex Torr. & A. Gray | Yes | 130000 | Dry |  |  |  |  | x |
| Orobanchaceae | *Pedicularis lanceolata* Michx. | Yes | 44000 | Wet | x | x |  |  |  |
| Plantaginaceae | *Penstemon grandiflorus* Nutt. | Yes ^b^ | 13000 | Dry |  |  |  | x | x |
| Poaceae | *Andropogon gerardii* Vitman | No | 10000 | Wet |  | x | x | x | x |
| Poaceae | *Bouteloua curtipendula* (Michx.) Torr. | No | 4000 | Dry |  |  | x | x | x |
| Poaceae | *Bromus ciliatus* L. | No | 5500 | Wet | x | x |  |  |  |
| Poaceae | *Eragrostis spectabilis* (Pursh) Steud. | No | 280000 | Dry |  |  |  |  | x |
| Poaceae | *Koeleria macrantha* (Ledeb.) Schult. | Yes ^a^ | 200000 | Dry |  |  |  | x | x |
| Poaceae | *Schizachyrium scoparium* (Michx.) Nash | No | 15000 | Dry |  |  | x | x | x |
| Poaceae | *Sorghastrum nutans* (L.) Nash | No | 11000 | Wet |  |  | x | x | x |
| Poaceae | *Spartina pectinata* Bosc ex Link | No | 6000 | Wet | x | x | x |  |  |
| Poaceae | *Sporobolus cryptandrus* (Torr.) A. Gray | No | 200000 | Dry |  |  | x | x | x |
| Ranunculaceae | *Thalictrum dasycarpum* Fisch. & Ave'-Lall. | No | 11000 | Wet |  | x | x |  |  |
| Rhamdaceae | *Ceanothus americanus* L. | Yes | 7600 | Dry |  |  | x | x | x |
| Rosaceae | *Rosa blanda* Aiton. | Yes | 3500 | Dry |  |  | x | x | x |
| Verbenaceae | *Verbena hastata* L. | Yes | 93000 | Wet | x | x | x |  |  |
| Verbenaceae | *Verbena stricta* Vent. | Yes | 25000 | Dry |  |  |  | x | x |
